# The Stress Response Is Adaptive in a Context- and State-Dependent Manner

**DOI:** 10.3390/cells14241957

**Published:** 2025-12-09

**Authors:** Harmen J. Krugers, Marian Joëls

**Affiliations:** 1 SILS-CNS, Swammerdam Institute for Life Sciences, University of Amsterdam, Science Park 904, 1098 XH Amsterdam, The Netherlands; 2 Center for Urban Mental Health, University of Amsterdam, 1019 XH Amsterdam, The Netherlands; 3 University Medical Center Groningen, University of Groningen, 9713 GZ Groningen, The Netherlands; m.joels@umcg.nl

**Keywords:** adaptation, memory, resilience, prediction, flexibility, metaplasticity, cell, behavior

## Abstract

A long-standing question in stress physiology is when and how the adaptive stress response can become maladaptive. In this narrative review primarily focusing on experimental studies in (male) animals, we argue that this is not merely a semantic matter but that—within limits—the organism’s response to an acute or chronic stressor and the interpretation thereof as adaptive or maladaptive very much depends on the context and the circumstances under which the response is studied, including the task configuration and the state of the organism, in relation to its recent and past stress history. We substantiate this by providing examples at the behavioral level. The behavioral findings can be understood from neurophysiological studies, which show that the recent and distant (stress) history of an organism shapes its brain function and plasticity. Consequently, renewed exposure to a challenging situation evokes a different cellular and circuit response than it would without this history, pointing to metaplastic changes. Overall, we argue that many of the stress-induced effects that are currently interpreted as maladaptive are, in fact, adaptive as long as the environment aligns with the circumstances predicted by the individual’s experience. Adaptation to novel circumstances will require (behavioral) flexibility. Understanding the relationship between metaplastic or state-dependent cellular responses on the one hand and flexible behavioral outcomes that foster adaptation on the other hand may contribute to developing interventions or preventing stress-related mental disorders.

## 1. Introduction

**Figure 1:**
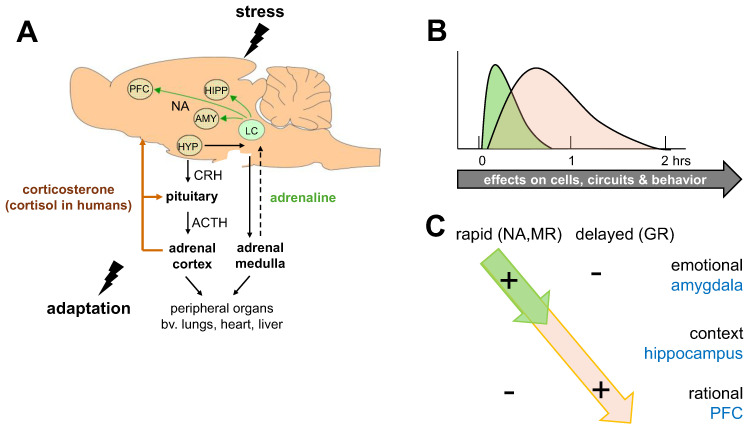
The bimodal stress response. (**A**) Schematic representation of two major systems activated after stress exposure: (1). The sympathetic nervous system, resulting in the release of (nor)adrenaline from the adrenal medulla; this indirectly feeds back on the brain, causing activation of noradrenergic (NA) projections originating in the locus coeruleus (LC). (2) The hypothalamus–pituitary–adrenal axis, which results in the secretion of corticosterone (or cortisol in humans) from the adrenal cortex. Corticosterone shuts down the peripheral tissue responses to stress as well as its own release, primarily at the pituitary level. The hormone also reaches cells in the brain. In some brain regions, such as the prefrontal cortex (PFC), amygdala (AMY), and hippocampus (HIPP), interactive actions of the two systems have been described. HYP = hypothalamus; CRH = corticotropin releasing hormone; ACTH = adrenocorticotropic hormone. (**B**) Cells in the brain are exposed to subsequent and partly overlapping waves of stress mediators, including noradrenaline (green) and corticosterone (orange). Consequently, cellular activity (and hence the circuits and behaviors in which these cells are involved) will change. In the case of corticosterone (and probably other stress mediators), the actions can last for hours, i.e., beyond the time window during which hormonal levels are elevated. (**C**) Whole-brain single-cell, neuroimaging, and behavioral studies support the existence of a bimodal stress response. In the short term (<15 min after stress), the salience network (most notably, the amygdala) is activated, which is linked to increased vigilance, attention, and emotional performance, with a focus on the survival of oneself and close ones. During this phase, higher cognitive functions are suppressed. Fast-acting monoamines (such as noradrenaline, NA), peptides, and corticosteroids via non-genomic pathways (involving the brain mineralocorticoid receptor, MR), play a prominent role directly after stress. At a later stage (>1 h after stress), genomic glucocorticoid receptor (GR)-dependent mechanisms prevail, which suppress emotional processing and, rather, promote circuits involved in context-dependent memory formation, rational decision-making, and altruistic choices, all of which prepare organisms for similar situations in the future.

Individuals are frequently exposed to (potential) perturbations of their mental or physical equilibrium as a result of perceived unpredictable and/or uncontrollable events (stressors). This is subjectively experienced as “stress”. Those exposed to stressors respond with an evolutionarily well-conserved bodily reaction, the stress response (see Figure 1A). Information about the potentially threatening events is quickly processed in cortical areas, and the resulting brain activity funnels through the hypothalamic paraventricular nucleus (PVN), causing a fast activation of the sympathetic system, with its main actor (nor)adrenaline, followed shortly thereafter by activation of the hypothalamus–pituitary–adrenal (HPA) axis, eventually resulting in the secretion of glucocorticoids from the adrenal cortex which primarily help to mobilize energy resources required for coping with the stressor. Corticosteroid hormones exert negative feedback on the stress-induced peripheral cell and tissue changes as well as (indirectly) on their own release at the level of the pituitary gland and the hypothalamus, so that circulating hormone levels gradually return to baseline. In addition to these central negative feedback sites, corticosteroid hormones—primarily cortisol in humans and corticosterone in most rodents—reach many brain nuclei where they induce, in concert with other stress mediators, a plethora of cellular and circuit effects [1], thereby altering the organism’s behavior and allowing it to cope with environmental challenges [2,3]. Different coping styles have been distinguished [4], including a proactive coping style involving escape behavior, aggression, and (active) avoidance, which is acquired as goal-directed instrumental behavior and can be extinguished when the goal cannot be achieved; and a reactive, more passive coping style, including submissive behavior and freezing, which lacks the impetus to address the source of the stressors [5].

While the stress response is generally considered adaptive, there are limits to its adaptive capacity. Especially in the face of severely traumatic events or cumulative adverse life events—particularly when these occur during the vulnerable developmental phase—the adaptive capacity may ultimately fall short. More generally, some individuals (be they humans or rodents) show behavioral responses under non-stressful and particularly under stressful conditions that appear inappropriate or ineffective in addressing the situation at hand. It is generally assumed that in these individuals, the stress response has become maladaptive.

A long-standing question in stress physiology has therefore been when and how the adaptive stress response can become maladaptive. Here, we will argue that—within limits—the organism’s response to an acute or chronic stressor and the interpretation of this response as adaptive or maladaptive very much depends on the context and the circumstances under which it is studied, including the task configuration and the state of the organism, in relation to its recent and past stress history. This is evident at the behavioral level and might be understood from the cellular and network effects in the brain seen after exposure to acute or chronic stressors. In our view, many of the stress-induced changes currently interpreted as maladaptive are, in fact, adaptive as long as the environment aligns with the circumstances predicted by the individual’s experience. To substantiate this view, we will first describe the bimodal cellular and behavioral responses to stress, and then highlight the importance of the test conditions for interpreting the observed behavior. We will attempt to understand how the latter can be explained at the cellular and circuit levels of the brain. The focus of this review will mainly be on results obtained in (male) experimental animal models; however, whenever relevant, we will also highlight parallel work in human subjects. In the final section, we will discuss the limitations of currently used experimental designs, and evaluate how our views relate to the concepts of vulnerability and resilience.

## 2. The Bimodal Stress Response

Any perceived threat to the mental or physical equilibrium of individuals triggers a cascade that rapidly releases adrenaline from the adrenal medulla and more slowly secretes glucocorticoids from the adrenal cortex (Figure 1A). As a consequence, peripheral organs as well as the brain will be exposed to sequential, partly overlapping waves of neurotransmitters, neuropeptides (like Corticotropin Releasing Hormone, CRH), and hormones such as corticosteroids that potentially alter cellular properties and hence the function of organs, including the brain (Figure 1B; [6]). In the following, we primarily focus on glucocorticoid actions in the brain, although the overall impact of stress involves many organs and multiple mediators that act in concert [1].

In the brain, glucocorticoids bind to high-affinity mineralocorticoid receptors (MR) and lower-affinity glucocorticoid receptors (GR) (for review see [7]). These are intracellular molecules with a distinct regional distribution that act as transcriptional regulators, of which the functional consequences generally are discernible with a delay of approximately 1 h. In addition, it has been recognized over the past decades that corticosteroids can exert actions via membrane-associated receptors, altering cellular properties within minutes (for review, see [8]).

Electrophysiological studies have shown that neurons in various brain regions exhibit specific responses to stress mediators, which occur in partially overlapping yet distinct time domains. For instance, in parvocellular neurons of the PVN, corticosterone can quickly suppress the release probability of glutamate-containing vesicles via a mechanism involving retrogradely transported endocannabinoids [9]. This is thought to contribute to the fast negative feedback of the HPA axis [10], which was already described decades earlier [11]. More slowly, the excitation-inhibition balance in this region is repressed via multiple inputs from surrounding regions (for review, see [12]).

In the hippocampus, corticosterone, within minutes and reversibly, increases the release probability of glutamate-containing vesicles via a mechanism that requires MR activation [13]. Single molecule imaging studies revealed that corticosterone—within the same time frame and via MRs—increases (i) the mobility of AMPA receptors, and (ii) activity-dependent retention of AMPA receptors [14]. More slowly, corticosterone, acting now via GRs, increases AMPA receptor mobility, the synaptic retention of AMPA receptors, and AMPA receptor-mediated synaptic transmission [15,16,17,18], while suppressing the transfer of steady input [19,20], thereby enhancing the signal-to-noise ratio. These persistent effects of corticosterone on AMPA receptor synaptic content require changes in NMDA receptor synaptic dynamics [21]. Functionally, this translates into a model whereby corticosterone via MR and GR activation rapidly yet persistently regulates the dynamics of hippocampal AMPA and NMDA receptors. At the circuit level, corticosterone enhances synaptic plasticity when hormone levels are elevated at or around the time of inducing plasticity which promotes well-described effects of glucocorticoids on memory consolidation [22,23,24,25,26]. The persistent effects of stress and corticosterone on AMPA receptor-mediated synaptic transmission may, via metaplastic effects, prevent further synaptic potentiation of already potentiated synapses, an effect that is well-described in the literature [27,28].

Corticosterone also rapidly alters excitatory (and inhibitory) synaptic transmission in the (basolateral) amygdala. Via MR activation, corticosterone enhances excitatory synaptic transmission by increasing the release probability of glutamatergic vesicles, an effect that is more persistent in the amygdala than in the hippocampus [29]. GABAergic synaptic transmission was shown to be reduced, involving the endocannabinoid CB1 receptor [30].

In the infralimbic cortex, corticosteroids rapidly reduce excitatory synaptic transmission, while leaving GABAergic transmission unaffected [31]. More slowly, stress was reported to persistently increase glutamatergic transmission in the prefrontal cortex, by a GR-dependent enhancement of AMPA and NMDA receptor-mediated synaptic transmission that underlies memory-enhancing effects of glucocorticoids [18,32].

Overall, these effects at the cellular and circuit level contribute to rapid activity-dependent changes in the hippocampus, amygdala, and, with a longer delay, in the prefrontal cortex, while persistently increasing storage of information at the synaptic level. In line with this, corticosteroids and stress exposure enhance memory formation via the regulation of excitatory synaptic receptors [18,22,32,33]. The changes in glutamate signaling may also relate to well-documented stress effects on impairing the retrieval of information as well as the acquisition of novel information (see further below).

Of note, the way in which these corticosteroid-induced changes at the cellular and circuit levels were established is hampered by several limitations. First, most studies have only examined the effects exerted by a single stress mediator on neurons (rather than glial cells) and within a single time domain. This is relevant because other stress-induced neuromodulators, such as noradrenaline, also regulate AMPA receptor trafficking and synaptic plasticity and enhance learning and memory [34,35]. This is even more relevant because the various stress mediators interact with one another’s actions: There are multiple examples of rapid effects by a particular stress mediator that prime neurons so that the neuronal response to subsequent waves of other mediators is altered (see, e.g., [8]). In fact, the combination of corticosterone and noradrenaline was found to optimally alter synaptic transmission [36], a finding that was also reported for memory consolidation ([37], see also below). Secondly, the electrophysiological recordings were usually carried out in a single area at a time, whereas in reality, circuits of interconnected regions underlie behavioral effects. Interestingly, a recent study examining the sequential effects of an inescapable foot shock on c-fos expression at the single-cell level across the entire brain essentially confirmed the above-described rapid and delayed activations in various brain regions [38]. The observations in rodents on sequential region-dependent activity also agree with findings in humans, using fMRI or PET imaging (see, e.g., [39]).

In agreement with the electrophysiological findings in rodents, behavioral studies in rodents and humans (the latter sometimes in combination with functional MRI recordings) showed that stress causes distinct behavioral changes depending on when the brain is probed relative to the stressor ([6,39,40]; summarized in Figure 1C). Thus, within 15 min after stress (or a rise in corticosteroid levels), individuals show increased appraisal of the situation at hand, vigilance, and focused attention on salient information; higher cognitive functions, by contrast, are temporarily suppressed. More generally, shortly after stress, individuals revert to relatively simple behavioral strategies, including those based on habit learning [41], which optimally promote short-term survival of oneself and close ones [42]. In the aftermath (>1 h later) of stress and rises in corticosteroid level, though, higher cognitive processes are promoted, allowing the individual (i) to choose a more flexible, context-related strategy, (ii) improve goal-directed behavior, and (iii) make more altruistic choices. Overall, the delayed effects of stress mediators help to (iv) rationalize experienced situations, and store relevant aspects of the context in which the stressor was encountered [43]. Of note, the distinct, time-dependent effects of corticosteroid hormones do not negate the importance of other stress mediators, such as monoamines, vasopressin, or CRH, particularly immediately after stress exposure.

Based on the distinct cellular and behavioral effects in the two time domains, we earlier proposed that the rapid MR- and delayed GR-dependent actions operate in a complementary manner, functioning as an on/off switch to coordinate circadian events, stress coping, and adaptation ([2]; see Discussion). A good balance between the two modes of action seems a prerequisite for optimal survival, not only in the face of an immediate threat but also to anticipate potential stressors in the longer term and to be better prepared for the future. Obviously, when the initial response falls short, inappropriate response selection may arise and be stored for future use. However, the reverse may also pose individuals at risk. If the rapid (relative to the delayed) response to stress dominates, individuals might become hyperaroused after stress exposure, a state that the delayed phase cannot sufficiently contain and that cannot be adequately rationalized or properly contextualized. A disbalance in the bimodal stress response may contribute to inadequate cognitive processing during daily stressors and thereby contribute to the development of psychopathology.

Clearly, to discern aberrations in the bimodal stress response, it is necessary to study brain activity and cognitive processing during both phases, which is rarely done. Studying individuals only directly after stress (as is the case in most reports) would reveal only one side of the coin and give an incomplete picture of the adaptive or maladaptive character of the response. Moreover, it is important to examine how stable or malleable adaptive responses are during recall, also in the longer term (see, e.g., [44]), as recall is an occasion when stored information may be interfered with by novel information [45].

## 3. The Relevance of the Circumstances During Testing

Whether the response to (acute or chronic) stress is adaptive or maladaptive depends, among other things, on the demand. Obviously, some circumstances are so intense (e.g., traumatic, life-threatening situations) that the adaptive capacity of the individual’s stress response may fail: The intense focus on sheer survival can disrupt the balanced response and thus introduce a substantial risk of maladaptation once the life-threatening situation is over. But often, the stress response—even after multiple or repetitive stressors—might seem to be maladaptive when tested under some circumstances yet completely adaptive under others.

We will here discuss two aspects that may contribute to the interpretation of the experimental results as (mal)adaptive: (i) the task selection, and therefore the context, in which an organism is tested; and (ii) the state of the individual in light of its recent or distant experiences—in particular its stress history. While we recognize that multiple factors have been identified as risk factors for maladaptation, including genetic background, sex, age, mitigating factors such as social support, and aggravating factors such as low socioeconomic status, we will not consider these factors here and refer readers to other reviews (e.g., [46],^47^,^48^,^49^) for details.

### 3.1. Relevance of Context and Experimental Design

Stress affects the link between context and stress-related information. For one thing, stress strengthens the degree (“the quantity”) to which a particular context is linked to information: re-exposure to that same context helps to recall the emotional (fearful) or neutral (safe) aspects of events, through both noradrenaline and corticosteroid-dependent mechanisms, in rodents (e.g., [34,50]) as well as humans [51]. Yet, stress also affects the *quality* of contextual memory. Thus, in fear conditioning or inhibitory avoidance tasks, noradrenaline was found to particularly increase memory precision, which may be effective in predictable (i.e., previously experienced) environments [52,53]. Conversely, post-training administration of glucocorticoids enhanced memory consolidation in a more generalized manner: rodents showed increased freezing or avoidance in a similar, yet different environment [54,55,56,57]. In principle, the latter is adaptive, as it enables individuals to prepare for future threats and potentially enhance alertness in unpredictable environments. Yet, excessive fear or memory generalization may result in maladaptive, inappropriately anxious behavior and disturbed declarative (memory) recall. This example illustrates that extending the task from a single to various contexts helps to better appreciate the (mal)adaptive nature of the individual’s response.

Another important aspect of interpreting behavior as adaptive or maladaptive relates to the degree to which individuals are allowed to adopt alternative strategies to solve the task. This is nicely illustrated by a series of experiments by Schwabe and colleagues [40,41,58,59,60,61], who used a task where rodents or humans were asked to solve a spatial memory task (finding an exit hole for rodents or locating a card for human subjects). In this task, subjects could employ a spatial strategy, using spatial cues in the environment, or a stimulus–response strategy (with a cue closely connected to the exit hole or card) to solve the task. In male rodents as well as human subjects, stress triggered a switch from using a spatial strategy to a stimulus–response strategy [41,58]. This switch required MR activation and was associated with a shift toward the use of striatal pathways [62,63]. If subjects had been allowed to use only the spatial strategy, the (wrong) conclusion might have been that stress impairs memory and that its effects are overall maladaptive. The reality turned out to be more complex, as organisms were actually able to solve this task under stressful conditions by reverting to an alternative strategy, allowing them to cope with the stressful situation at hand. Importantly, selecting a different strategy may come at a cost, as stimulus–response learning is relatively rigid and may fall short when the situation is less predictable and demands greater flexibility, as was, e.g., observed after chronic stress [64].

These examples serve as mere illustrations that the experimental design is crucial for understanding the adaptive nature of stress on behavior. We highlighted two (among many other) aspects: (i) the context in which animals are tested (an external factor, linked to predictability and expectancy); and (ii) the capacity of animals to switch behavior depending on the demand (an internal factor, related to flexibility). Obviously, there is an urgent need to employ more elaborate tasks to fully appreciate the coping strategies of individuals—rodents and humans alike. Additionally, more detailed analysis methods will aid in better interpreting behavioral outcomes. In this respect, the use of machine-learning approaches to measure complex behavioral expressions and social dynamics, which is critical for coping over time, is an interesting development [65,66,67,68,69].

### 3.2. Current State of the Individual in Relation to Its Recent or More Distant History

The state of the individual at the time of testing is relevant when determining the adaptive nature of a stress response. This is especially pertinent when the test situation is intrinsically arousing or stressful. This was nicely demonstrated in the Morris water maze, where rodents use spatial cues in the (remote) environment to escape from the water and climb on a non-visible platform; during the task and the subsequent consolidation phase, they form a cognitive map which they can use to navigate on future occasions. The water temperature and rise in glucocorticoid levels during acquisition were shown to be critical factors for retention of the task [22,70]. Thus, lowering the water temperature from 25 to 19 °C during acquisition increased circulating corticosterone levels and improved remote spatial memory formation. These and similar studies (e.g., reporting on fear conditioning) led to the notion that stress can promote learning and memory formation (for reviews [24,71]), which is adaptive when individuals encounter a comparable situation in the future. In the example of the Morris water maze, the aroused state forms an integral part of the test situation: Corticosteroid levels must be high during the consolidation phase for retention to be promoted. By contrast, if individuals are stressed before retrieval (out-of-context), the recall of already stored information was generally found to be impaired [23,71,72]. Impaired retrieval of earlier learned information could be maladaptive if the earlier stored information is essential for the organism (and its survival). However, it could also be adaptive when prioritizing salient information linked to the novel stressor is imperative.

The state of the animal during testing is also relevant when it has a distant history of stress. This is most evident in the case of learned helplessness, where rodents transfer their experience from an earlier stressful situation to new stressors [73]. The earlier stressor may leave animals seemingly unaffected under non-stressful conditions, but a clear phenotype emerges when they are examined during a novel stressor. Their passive coping style represents an innate response to the adverse condition, yet they have learned from earlier experiences to overcome this by exerting control over the situation. As such, it is somewhat reminiscent of rodents’ behavior in the Porsolt swim test, where immobility also represents an (innate) default mode in order to conserve energy and survive in an inescapable situation [74]. The acquired immobility response is retained 24 h later, and one may assume that animals have “learned” that struggling is ineffective. The presumed beneficial effect through learning of earlier stress exposure on how to deal with arousing or stressful conditions later on also forms the basis for the concept of stress inoculation in animals and humans [75,76].

A special case is when earlier stress occurred during the first postnatal weeks of rodents. During this developmental period, the brain is highly sensitive to stressful experiences, which shape the brain and behavior in anticipation of a predicted environment, in part through epigenetic changes with long-lasting consequences (for reviews, see [77,78]). A crucial element of many early-life stress models is the unpredictability regarding food supply and care, particularly when the mother shows erratic behavior [79]. Extreme situations aside, we and others have argued that animals will show an adaptive phenotype when tested in adulthood under conditions predicted by their earlier life history. Conversely, their behavior may seem maladaptive when later-life conditions do not match the prediction [80,81,82,83]. This notion of the so-called match-mismatch theory was based on the observation that stressful experiences early in life generally reduce adult learning and memory processes under relatively non-stressful conditions, while memory formation in tasks that are inherently stressful, such as contextual fear conditioning, is promoted [84], although the latter has not been universally confirmed [85].

Interestingly, early life adversity can result in perseverance of behavior in adulthood [86]. While this can be interpreted as a lack of flexibility, another interpretation could be that animals with a history of early life adversity show behavior guided by what was particularly “meaningful” during early life. This emphasizes the relevance of studying the ability to change behavior in adulthood and the expression of behavioral flexibility. Memory and predictability are likely to play a relevant role here: What has been learned earlier, and is this in line with what was remembered? And can behavior be adjusted according to what is required? The latter likely requires a process of memory reactivation and reconsolidation to express behavioral flexibility, possibly depending on “prediction errors” [87].

**Figure 2:**
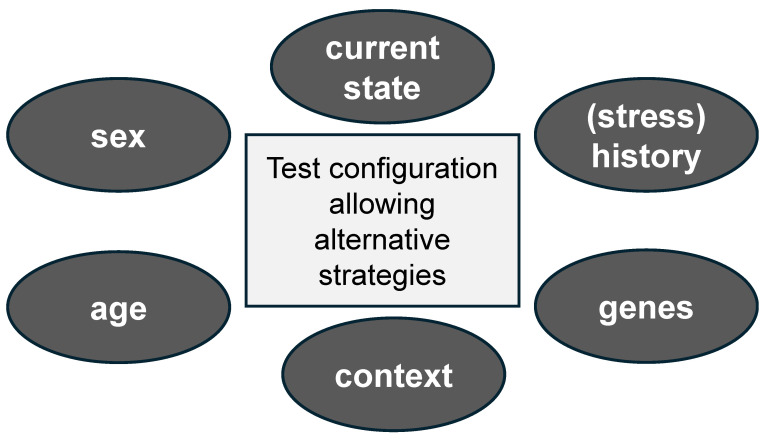
Insight into the adaptive nature of an animal’s behavior requires test configurations that allow more complex behavioral repertoires. Many more factors will determine the eventual outcome, including (but not restricted to) the sex and age of the organism, contextual elements (outside the test configuration, such as housing condition, social environment, etc.), the organism’s genetic background, its life history, and its state (e.g., aroused) during testing.

In conclusion, animals may show appropriate responses under non-stressful conditions, but not when exposed to a stressful task, or vice versa. There is a learning component in this, in the sense that organisms learn to adapt their behavioral responses to environmental challenges. A full appreciation of the extent to which a response is (mal)adaptive can therefore only be obtained when organisms are observed in various states, while taking their recent and remote history into account (Figure 2).

## 4. Similarities at the Single-Cell and Network Level

How can the relevance for behavioral outcome of a predictable context and the state of the animal—and hence the adaptive nature thereof—be understood at the level of the underlying neuronal substrate? Although the available literature is relatively scarce, we provide several examples from our own work where the predictable context, in light of the animal’s recent or remote (stress) history, in combination with its current state, determines the cellular or network function.

### 4.1. Recent Stress Experience, Synaptic Plasticity, and Memory

**Figure 3:**
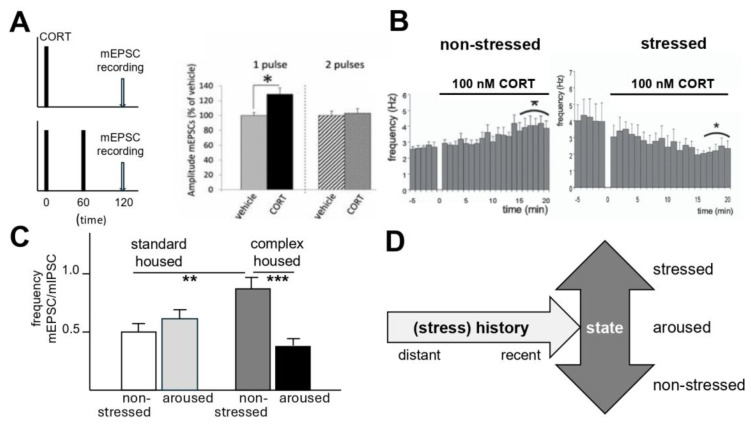
Examples of cellular metaplasticity in relation to (earlier) changes in corticosteroid level. (**A**) Neurons respond (120 min) after a single 20 min pulse of (100 nM) corticosterone (CORT) with an increased amplitude of the miniature excitatory postsynaptic current (mEPSC), which represents the cell’s response to one spontaneously released glutamate-containing synaptic vesicle. If the cell is exposed to a second CORT pulse 60 min after the first, which mimics the normal ultradian rhythm, the mEPSC amplitude remains stable, thus ensuring that the entire range of the cell’s responsivity to incoming information is intact. *: *p* < 0.01. From ref. [88]. (**B**) Application of 100 nM CORT increases within 10–15 min the mEPSC frequency of principal basolateral amygdala cells. This enhancement is sustained. If mice are exposed to a restraint stressor and tested >1 h later for their cellular response to 100 nM CORT, basolateral amygdala cells show a *decrease* in mEPSC frequency. *: *p* < 0.05. ref. [29]. (**C**) The frequency of mEPSCs relative to that of inhibitory postsynaptic currents (mIPSCs)—which is sometimes used as a proxy for the excitation-inhibition ratio of a cell—is increased in infralimbic prefrontal layer2/3 pyramidal cells of mice exposed to 10 days of complex housing in adulthood relative to standard-housed mice, but only when both groups were tested under non-stressed conditions. By contrast, complex-housed mice that were mildly aroused shortly before slice preparation showed a *decreased* excitation-inhibition ratio. **: *p* < 0.01; ***: *p* < 0.001. From ref. [89]. (**D**) Collectively, these examples illustrate that the full impact of a recent or distant history of stress (or ultradian CORT pulses) on cellular activity can be best appreciated when tissue is obtained not only from animals under rest but also when they are challenged. (**A**): Reprinted/adapted with permission from ref. [88], copyright 2014, PNAS; (**B**): Reprinted/adapted with permission from ref. [29], copyright 2010, PNAS: (**C**): Reprinted/adapted with permission from ref. [89], copyright 2025, Elsevier.

As discussed before, there is substantial evidence that stress hormones like corticosterone and other neuromodulators alter dorsal hippocampal glutamatergic synaptic transmission and memory. Via MRs, these hormones prime synapses to rapidly express synaptic plasticity. Via GRs, synaptic plasticity can be maintained via the insertion of AMPA receptors, providing a model that supports memory consolidation. Enhanced synaptic transmission may, via metaplastic mechanisms, impair further potentiation, which could explain how stress prevents interference of earlier learned information by novel information. Such hippocampal metaplasticity may also occur due to daily circadian and ultradian variations in the corticosterone level [88]. Thus, a single (ultradian-like) pulse of corticosterone caused synaptic enrichment of glutamate receptors and increased responses to spontaneously released glutamatergic vesicles, collectively—as expected—hampering the ability to induce synaptic long-term potentiation subsequently (see Figure 3A). Interestingly, a second ultradian-like pulse one hour later completely normalized glutamate transmission, thus restoring the plastic range of the synapse. This metaplastic phenomenon may ensure that hippocampal glutamatergic synapses remain fully responsive and able to encode new stress-related information around the time that daily activities start.

Stress also alters basolateral amygdala (BLA) function via metaplastic mechanisms. Following brief corticosterone administration in non-stressed mice, BLA neurons exhibited prolonged enhancement of glutamatergic transmission, suggesting an extended window for encoding of emotional information ([29], Figure 3B). In animals with a recent history of stress (i.e., over the past hours), the enhanced BLA transmission was reversed via a GR-dependent *reduction in glutamatergic* transmission. The latter can (similar to the metaplasticity in the hippocampus) be interpreted as an effective way to protect the encoding of emotional information from interference by novel, unrelated events.

### 4.2. Chronic Stress, Current State, and Excitability

Various studies have investigated the role of chronic exposure to stress on excitability in the prefrontal cortex. Three weeks of chronic unpredictable stress reduced excitability in pyramidal cells in the infralimbic and prelimbic cortex, while leaving the excitation-inhibition balance unaltered in parvalbumin interneurons [90,91]. The underlying neuronal mechanisms may be region-specific, as chronic stress exerted its effects in the prelimbic region via an enhanced inhibitory drive from interneurons as opposed to a reduced excitatory drive from interneurons in the infralimbic region [91].

Interestingly, housing male mice in a complex (stressful) environment for 10 days in adulthood *enhanced* the excitation-inhibition balance in the infralimbic cortex of undisturbed animals examined at the circadian trough ([89]; Figure 3C). However, the excitation-inhibition balance was *reduced* (comparable to [90,91]) in mice that showed signs of mild arousal, which indicates a state-by-conditions interaction. Whether and how this relates to adaptive behavioral responses in a complex environment requires further investigation.

### 4.3. Early Life Stress, Predicted Conditions, Synaptic Plasticity, and Learning

In most rodents, the presence of the dam is critical for nutrition and care of the offspring. Variation in maternal care programs brain function and behavior, determining plasticity and performance of the offspring later in life. Studies by Michael Meaney and colleagues have shown that the offspring from mothers that express high levels of licking and grooming (LG) during the first postnatal week have better spatial memory and synaptic plasticity than offspring from low LG mothers, although differences exist between the dorsal and the ventral hippocampus [81,92,93]. Synaptic plasticity effects reminiscent of those seen in offspring of low LG mothers have been reported in other models of early life stress, such as a model based on housing the dams and their offspring with limited bedding and nesting material, which induces fragmented and unpredictable maternal care [94,95]. These effects of early life adversity on behavior are related to alterations in NMDA receptor function [96,97]. Interestingly, the deficits in synaptic plasticity seen in (adult) offspring from low LG mothers could be restored by acute administration of corticosterone in adulthood, while in high LG offspring, corticosterone was ineffective or even resulted in impaired synaptic plasticity [81]. Also, the NMDA-AMPA response ratio that was reduced in the LBN model could be restored by acute corticosterone treatment [98]. These electrophysiological studies at the level of hippocampal cells and circuits argue that experiences early in life lastingly program plasticity at the level of cells and circuits, resulting in relatively ineffective synaptic plasticity (in conjunction with less effective learning under non-stressful conditions) in those who were exposed to adverse conditions early in life. The critical twist in the story is that those exposed to early adversity show very effective synaptic plasticity (and learning) later in life when tested under stressful conditions, emphasizing that the distant stress history of the organism and its current state are both important to understand the adaptive (or maladaptive) nature of the response. This agrees with Diniz and Crestani [99], who argued that plasticity mechanisms and expression of plasticity can be upward or downward, but that both directions are necessary for behavioral flexibility and adaptation. An inability to express plasticity when needed may result in an inability to express flexibility and to alter behavior in response to the demand.

All in all, these examples illustrate that the recent and distant (stress) history of the organism shapes its brain connectivity such that renewed exposure to a challenging situation evokes a different cellular and circuit response than without this history (summarized in Figure 3D). The available data support the notion that there are considerable regional differences in these (meta)plastic changes, which to date have been explored only in a cursory manner. This warrants more detailed investigations, not only in isolated brain areas, but also in interconnected networks that contribute to behavioral outcomes. Also, a more dedicated investigation of the underlying molecular mechanisms, probably involving epigenetic programming (and likely in a sex-dependent manner, see, e.g., [100,101]), requires more attention. Moreover, a direct and causal relationship between metaplastic or state-dependent cellular responses, on the one hand, and behavioral consequences, on the other, has not yet been firmly established. For now, the physiological experiments mentioned above merely point to putative neuronal mechanisms underlying the context- and state-dependency of (mal)adaptation. There is a need for experiments that test the performance of animals in various contexts that do or do not align with what the animals can predict based on their experience, and also allow subsequent testing to investigate whether and how animals have learned, can express flexibility, and adjust their behavior in response to demands. Ideally, one would apply tools to conduct behavioral phenotyping and investigate the behavioral repertoire that animals use to perform, and combine this in vivo with approaches that examine the underlying neurobiological substrates, including synaptic properties, plasticity, and activity markers in ensembles of neurons. Once biomarkers of plasticity have been identified, intervention strategies can be employed with optogenetics or chemogenetics to detect causality between cellular plasticity and adaptive behavioral flexibility.

## 5. Concluding Remarks

In the parable of the blind men and the elephant, the men—each touching a different part of the elephant—all draw different conclusions about the nature of the animal they explore: It is a snake (the trunk), a tree (the leg), a fan (its ears), a rope (its tail) etcetera. By studying the various body parts in isolation, they miss the bigger picture. In our view, the conclusion whether particular responses to acute or chronic stress are adaptive or maladaptive often suffers from the same shortcomings. By (i) only concentrating on effects directly after stress (i.e., within minutes, not taking the delayed responses into account that are only seen >1 h later); (ii) by using simple behavioral tasks that usually probe just a few cognitive domains and leave individuals little room for alternative strategies, with the additional caveat of limited behavioral interpretations; and (iii) by testing animals (or humans) only under specific conditions—e.g., under rest and not an aroused state; or at the inactive and not the active phase of the day—one could inadvertently conclude that the individual’s behavior is maladaptive, while varying the task or testing conditions might lead to different conclusions. Here, we have highlighted only a few of the variables that may limit our insights, but no doubt, there are many more; we, too, have our blind spots. For instance, an essential element currently not discussed is that nearly all observations and conclusions are based on male organisms. The adaptive or maladaptive nature of the stress response in females warrants dedicated investigation and may yield different insights.

We argue that, apart from rather extreme conditions such as severely traumatic experiences, the stress response is, in principle, adaptive in a context- and state-dependent manner, i.e., the system is geared toward survival in both the short term and the longer term, and within the predicted circumstances. The bimodal stress response is ideally equipped to meet these demands and functions well, as long as the two modes are in balance. When the demands shift, for example, in the face of chronic (early-life) stress, gradual changes at the cellular and circuit levels may occur, disrupting the balance of the bimodal stress response. This is not necessarily a bad thing, as it prepares the organism and its system for comparable conditions later on. For example, chronic stress changes neurogenesis [102] and CA3 dendritic characteristics [103], which supposedly does not hamper the processing of “simple” information. Yet, it comes at the cost of reduced contextual discrimination and cognitive flexibility [104]. When the environmental context aligns with the predicted demands, even the effects of chronic stress can be considered adaptive [105]. However, they may become maladaptive when the context changes and the situation requires more flexibility.

**Figure 4:**
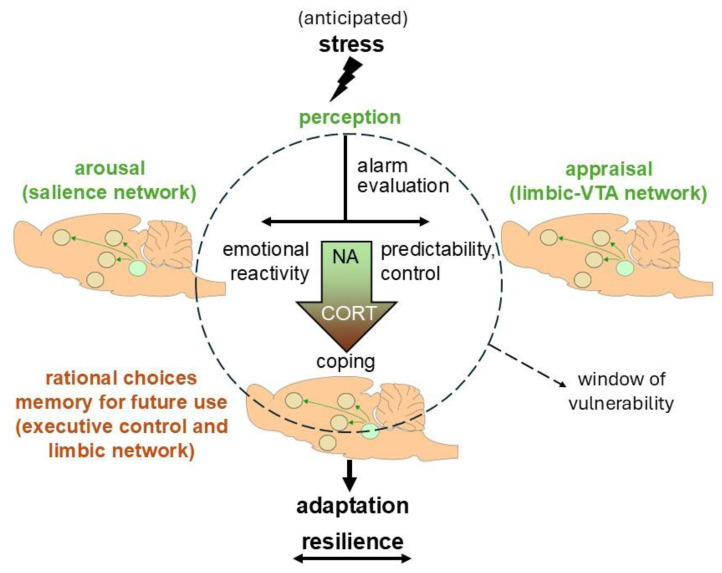
The bimodal stress response in relation to vulnerability and resilience. Incoming (potentially anticipated) stressful information is first perceived. This is followed by a phase that is characterized by quick memory retrieval, risk assessment, and response selection. Initially, the response primarily involves mediators of the autonomic nervous system (like noradrenaline (NA); green color), in collaboration with neuropeptides like CRH, or even non-genomic actions via corticosterone (CORT). Later, genomic effects via CORT predominate (orange color). The joint actions involve allocation of energy resources to circuits in need during stress coping and adaptation, and promote energy storage at rest. At a later stage (>1 h after stress exposure), CORT also promotes better performance of the executive control network and memory of the situation for future use. The actions exerted by CORT on information processing are conditional and time-dependent, and should therefore be considered in the context of CRH/vasopressin-driven hypothalamus–pituitary–adrenal axis, sympathetic, and behavioral reactivity in concert with multiple dedicated signaling cascades. Altogether, the response can lead to successful coping with the situation at hand. Organisms are particularly vulnerable when their earlier experience needs to be updated by newly learned adaptive responses (indicated by the striped circle). Those who are successful in flexible adoption of behavioral strategies are considered to be resilient. Resilience is thus not a static entity, but, rather, a dynamic process, here depicted by the sliding arrow at the bottom. VTA: Ventral Tegmental Area.

How do these statements about predictability and context-dependency of adaptation and maladaptation relate to the concepts of resilience and vulnerability, respectively? In general terms, resilience is defined as the ability to maintain mental and physical health during and after times of adversity [106]. This concept was adopted when it became evident that, although chronic (early) life adversity is an acknowledged risk factor for the development of psychopathology (see recent meta-analyses by, e.g., [107,108,109]), many individuals experiencing such adverse events stay healthy. Resilience involves active learning through positive reinforcement of behavioral coping strategies, recognition of safe situations, and a sense of controllability [110]. Interestingly, developmental psychologists have noted that the apparent resilient nature of specific (learned) behaviors is context-dependent, in other words, that they are efficient in some situations but not in others (reviewed in 111). They have pointed out that learning through experience about environmental controllability leads to a shift from negative controllability appraisal, pronounced stress reactivity, and inflexible, reactive coping styles to a more positive controllability appraisal, a higher sense of agency, less pronounced stress responses, and more flexible, proactive coping patterns [111]. This has led to a redefinition of resilience as a behavioral and physiological response that is flexible and adaptable across various situations and developmental stages [112]. The introduction of this non-static adaptability aspect is reminiscent of the shift from (fixed) homeostatic to (dynamic) allostatic setpoints in the field of stress physiology [113]. The redefinition of resilience implies that most individuals can overcome the context-dependency of adaptive responses through continuous learning. However, learning takes time, and thus, there always remains a window of vulnerability when circumstances do not match the expectation (see Figure 4). Moreover, there is a risk in the decoupling of learned information from its original context.

In humans, numerous factors contribute to an individual’s resilience and ability to cope with adversity. Some of these factors are biological, such as a person’s genetic background, brain architecture, or hormonal systems. Others are psychological, such as personality traits, coping strategies, or appraisal style. Also, the cultural and social environment plays a significant role, e.g., in biobehavioral synchrony [111] and via socioeconomic status (see, e.g., [47]). Variations in all of these factors and their interactions explain why only a minority of those exposed to adversity are at risk of developing psychopathology.

Resilience has also been extensively studied in animal models (reviewed in [114,115,116]). These models have the advantage that many of the “confounding” factors in humans can be controlled for. For instance, most labs use inbred mouse strains, minimizing genetic variation. The housing conditions are kept as stable as possible, too. Nonetheless, some mice exhibit a resilient phenotype in the face of (early life) adversity, while others display a vulnerable one, as assessed by a sucrose-preference test. One may wonder about the origin of this variation, given the relative homogeneity of the population. Most likely, the observed inter-individual variation is explained by variables that are not (and cannot be) controlled for, e.g., distinct variations in maternal care toward individual pups, not only between litters [81] but even *within* litters [117]. Moreover, it has been proposed that some individuals are more vulnerable to environmental factors than others, for better and for worse [118]. If so, slight variations in life history, e.g., with respect to maternal care, could be amplified in these vulnerable individuals, leading to a more variable phenotype in adulthood [119], with relatively more extreme phenotypes (either vulnerable or resilient) at the cost of the “average” group [120]. On the face of it, the extreme phenotypes, particularly the vulnerable ones, may seem maladapted; however, in reality, they are highly adapted in light of the predicted environment [121]. This supports the central message of our review, i.e., full appreciation of brain function and behavior as adaptive or maladaptive requires (i) taking cellular, behavioral, and neuroendocrine (e.g., feedback resistance) function during both phases of the stress response into account, as well as (ii) the state of the animal and (iii) the testing conditions. This was also pointed out by others [122,123], who stated that the current literature on adversity and resilience in animals is based on a limited set of read-outs, relatively simple behavioral tasks, and few life stages.

In conclusion, the discussion on adaptive or maladaptive brain function and behavior in response to stress is more than just a semantic issue. It may seem self-evident from an evolutionary perspective that the stress response is inherently adaptive, but we argue that this depends on the context. This necessitates testing conditions that consider this context in relation to the state of the organism, and the possibility of studying the ability of individuals to employ an extensive and flexible repertoire of behavioral manifestations.

## Data Availability

No new data were created. The review is based on articles derived from databases of literature on biomedical sciences and life sciences.
